# Machine learning tools for deciphering the regulatory logic of enhancers in health and disease

**DOI:** 10.3389/fgene.2025.1603687

**Published:** 2025-08-13

**Authors:** Spyros Foutadakis, Vasiliki Bourika, Ioanna Styliara, Panagiotis Koufargyris, Asimina Safarika, Eleni Karakike

**Affiliations:** ^1^ 4th Department of Internal Medicine, Medical School, National and Kapodistrian University of Athens, Athens, Greece; ^2^ Neonatal Unit, First Department of Pediatrics, National and Kapodistrian University of Athens, Athens, Greece; ^3^ Department of Obstetrics and Gynaecology, School of Medicine, University of Patras, Patras, Greece

**Keywords:** deep learning, enhancers, genomics, machine learning, sepsis, transcriptional regulation

## Abstract

Transcriptional enhancers are DNA regulatory elements that control the levels and spatiotemporal patterns of gene expression during development, homeostasis, and pathophysiological processes. Enhancer identification and characterization at the genome-wide scale rely on their structural characteristics, such as chromatin accessibility, binding of transcription factors and cofactors, activating histone modifications, 3D interactions with other regulatory elements, as well as functional characteristics measured by massively parallel reporter assays and sequence conservation approaches. Recently, machine learning approaches and particularly deep learning models (Enformer, BPNet, DeepSTARR, etc.) allow the prediction of enhancers, the impact of variants on their activity and the inference of transcription factor binding sites, leading, among others, to the construction of the first completely synthetic enhancers. We present the above computational tools and discuss their diverse applications towards cracking the enhancer regulatory code, which could have far-reaching ramifications for uncovering essential regulatory mechanisms and diagnosing and treating diseases. With an emphasis on sepsis, a leading cause of morbidity and mortality in hospitalized patients, we discuss computational approaches to identify sepsis-associated endotypes, circuits, and immune cell states and signatures characteristic of this condition, which could aid in developing novel therapies.

## 1 Introduction

Transcriptional enhancers are DNA regulatory elements that control the levels and spatiotemporal patterns of gene expression during development, homeostasis, and pathophysiological processes (Banerji et al., 1981; Agelopoulos et al., 2021; Sur and Taipale, 2016; Rickels and Shilatifard, 2018; Furlong and Levine, 2018). Enhancers regulate transcription irrespective of orientation to the Transcription Start Site (TSS) (Banerji et al., 1981), can act over large genomic distances exceeding 1 Mbp (Lettice et al., 2003; Long et al., 2020) and can skip their closest gene in the linear DNA sequence to regulate distal genes (Kessler et al., 2023; Chen et al., 2024). There are two main models regarding the architectural organization of enhancers: the enhanceosome and the billboard model (Arnosti and Kulkarni, 2005; Jindal and Farley, 2021). The enhanceosome operates with a high degree of cooperativity between enhancer-bound proteins, with the exact organization of transcription factor (tf) binding sites being crucial for enhancer output. The archetype enhanceosome is that of the interferon beta, induced following viral infections (Thanos and Maniatis, 1995). Nevertheless, enhanceosomes are rather rare and have been described for a limited number of cases involving mainly cytokine genes (Giese et al., 1995; Jindal and Farley, 2021). On the other hand, the billboard model posits that the binding sites are flexibly arranged and the bound proteins act as an ensemble interacting independently with their targets (Kulkarni and Arnosti, 2003; Jindal and Farley, 2021).

In the following sections, we present the main wet-lab methodologies to identify enhancers at the genome-wide scale (Catarino and Stark, 2018) and characterize their regulatory grammar (Jindal and Farley, 2021) and 3D interaction networks (Uyehara and Apostolou, 2023). We also describe booming machine learning algorithms to aid the above tasks towards a better understanding of pathophysiological conditions.

## 2 Enhancer identification at the genome-wide scale

**FIGURE 1:**
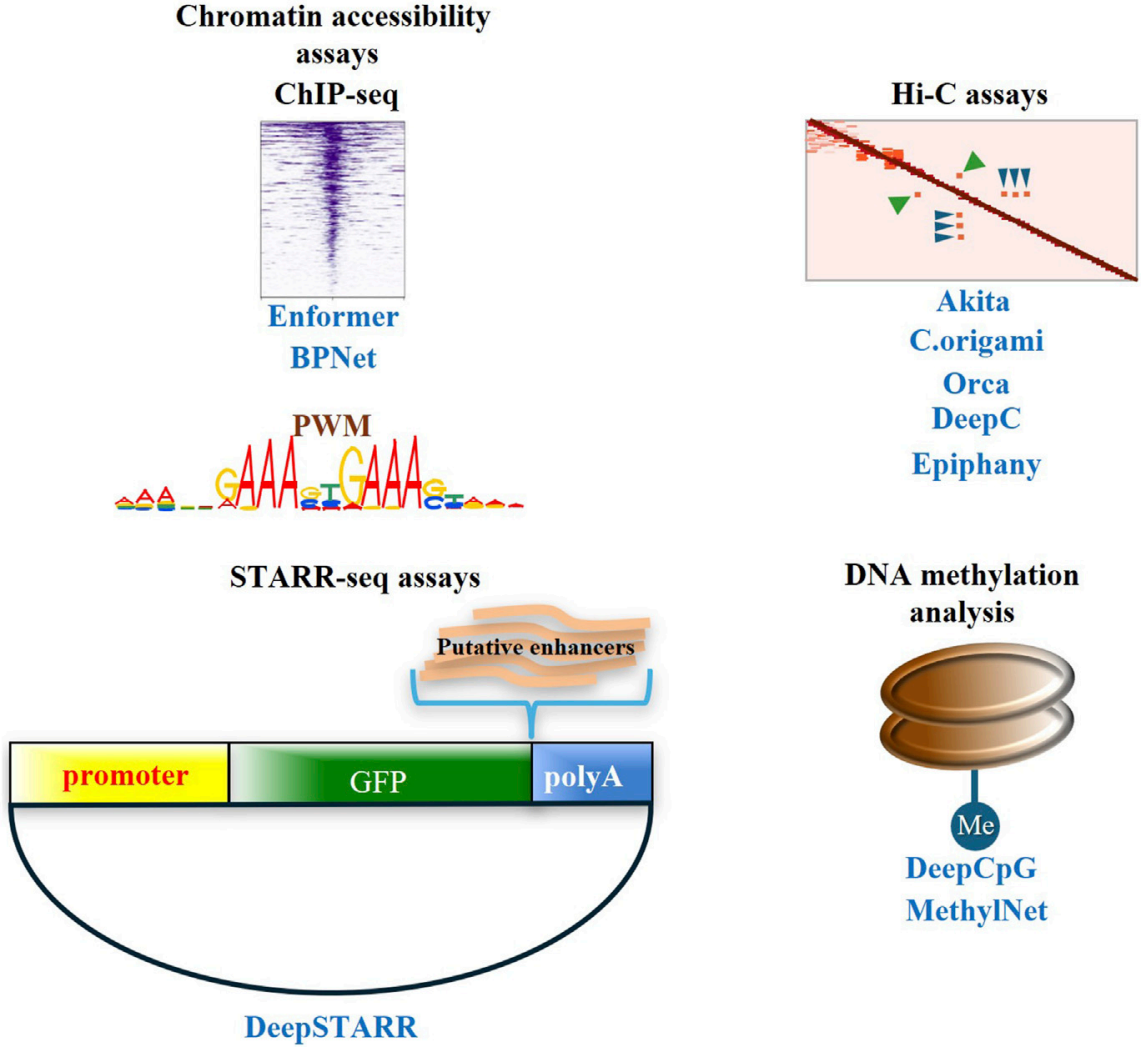
Depicted in black are the main (epi)genomics methodologies for studying enhancers, such as chromatin accessibility assays (ATAC-seq, DNaseI-seq) and ChIP-seq to identify open chromatin regions and the presence of histone modifications and tf and coactivator binding, respectively. Moreover, assays such as Hi-C are used to study the 3D organization of the genome, while massively parallel reporter assays like STARR-seq are used to examine the *bona fide* enhancer potential of putative regulatory regions. Depicted in blue are selected state-of-the-art deep learning algorithms trained with datasets produced through the above methodologies and used to identify enhancers, predict crucial motifs and assess the effect of variants on their function.

Enhancer identification and characterization at the genome-wide scale rely on their structural characteristics, such as chromatin accessibility (Buenrostro et al., 2013; Marinov et al., 2023; John et al., 2013; Vierstra et al., 2020), binding of transcription factors and cofactors (Robertson et al., 2007; Lambert et al., 2018), activating histone modifications (Heintzman et al., 2007; Calo and Wysocka, 2013; Barral and Déjardin, 2023) and DNA hypomethylation (Angeloni and Bogdanovic, 2019) (Figure 1). More specifically, chromatin accessibility denotes the regulatory potential of a DNA sequence and is measured by popular assays such as DNaseI-seq (John et al., 2013; Vierstra et al., 2020) and ATAC-seq (Buenrostro et al., 2013; Marinov et al., 2023). The combinatorial presence of histone modifications on chromatin constitutes a regulatory code (Kouzarides, 2007), with the genomic distribution of individual modifications usually measured with the ChIP-seq technology (Heintzman et al., 2007; Fadri et al., 2024). Histone modifications associated with active enhancers include H3K27ac and H3K4me1, while H3K27me3 and H3K9me3 are repressive marks (Heintzman et al., 2007; Calo and Wysocka, 2013; Barral and Déjardin, 2023). Most importantly, the binding of transcription factors and coactivators such as Med1 and CBP/P300 and other members of the transcription apparatus are also hallmarks of active enhancers (Catarino and Stark, 2018). High levels of the above enhancer markers are found in long stretches of enhancer DNA termed super-enhancers or stretch-enhancers that play crucial roles in the control of cell identity and disease-related genes (Hnisz et al., 2013; Whyte et al., 2013; Parker et al., 2013). The above epigenomics methodologies are frequently paired with whole transcriptome measurements using RNA-seq (Mortazavi et al., 2008; Deshpande et al., 2023) and the integration of the complementary multiomics methodologies provides a more holistic picture of the regulatory mechanisms that govern transcriptional programs.

Until recently the above (epi)genomics methodologies were only applied in bulk cell populations, thus providing only an ensemble of averaged signals, although it is well established that there is pervasive heterogeneity and stochasticity across systems, meaning that each individual cell inevitably has a unique profile (Eling et al., 2019). In the last decade, technological advances have ushered in the era of single-cell genomics with technologies such as single-cell RNA-seq (Tang et al., 2009; Klein et al., 2015), single-cell ATAC-seq (Buenrostro et al., 2015), simultaneous measurement of different modalities (Baysoy et al., 2023) and even spatial methodologies (Moses and Pachter, 2022; Deng et al., 2022), allowing the study of individual cells (Heumos et al., 2023).

All the above structural characteristics of enhancers are just convenient proxies for enhancer identification and do not prove that a certain DNA sequence holds true enhancer potential. The classic method to measure enhancer activity is the reporter gene assay, with the genomics-based derivatives STARR-seq (Arnold et al., 2013) and MPRA (Melnikov et al., 2012) allowing the massively parallel measurement of the enhancer potential for thousands or even millions of DNA sequences (Inoue and Ahituv, 2015). A limitation of the above methodologies is their episomal nature, measuring the activity of putative enhancers outside their native chromatin environment. The random genomic integration of the tested regions with the variant MPRA technology termed lenti-MPRA (Inoue et al., 2017) alleviates this problem to a certain extent, but nevertheless it does not allow the measuring of enhancer activity at the endogenous locus. An approach to measure enhancer activity at the native chromatin environment is the detection of enhancer RNAs (eRNAs), long non-coding RNAs that are produced from transcription of enhancer sequences, usually in a bidirectional fashion (Kim et al., 2010; Sartorelli and Lauberth, 2020). Nevertheless, eRNAs are of an unstable nature, necessitating the application of nascent RNA sequencing approaches such as GRO-seq (Core et al., 2008) and PRO-seq (Mahat et al., 2016) for their detection. The ultimate test to prove the activity of an enhancer and identify its target gene(s) is the disruption or mutation of its sequence with simultaneous measurements of its effect on the expression levels and chromatin environment of nearby genes and regulatory elements, respectively. This can be achieved with CRISPR-based methodologies such as CRISPR-interference (Fulco et al., 2016) that can silence an enhancer, CRISPR-mediated saturation mutagenesis (Canver et al., 2015) and even CRISPR-mediated activation of enhancers (Heidersbach et al., 2023). The above experimental methodologies to detect enhancers are complemented by DNA sequence conservation approaches to examine enhancer evolution across species (Siepel et al., 2005; Villar et al., 2015).

Large national and international consortia like the ENCODE (ENCODE Project Consortium, 2012), the Roadmap Epigenomics (Roadmap Epigenomics Consortium, 2015), the International Human Epigenome Consortium (Stunnenberg et al., 2016) and the Genotype-Tissue Expression Project (GTEx Consortium, 2013), as well as individual labs, have produced thousands of datasets that are deposited at public repositories such as the Gene Expression Omnibus (Clough et al., 2024) and the European Nucleotide Archive (Yuan et al., 2024) and are freely available for re-analysis and as training material for machine learning applications discussed in a following section.

## 3 Three-dimensional genome organization and enhancer communication

Vertebrate genomes, apart from the linear DNA sequence, are organized in the three-dimensional space of the nucleus with important ramifications for transcription regulation, DNA replication, and genome integrity (Misteli, 2020). The main approaches to studying 3D genome organization include Hi-C-based methodologies (Rao et al., 2014) or super-resolution microscopy (Boettiger and Murphy, 2020). The higher organizational unit of the genome is the chromosome compartment, with compartment A containing mainly active DNA regions, while compartment B hosts inactive regions (Rao et al., 2014). Although older studies produced rather sparse Hi-C datasets that examined compartments at the Mbp scale, recent efforts using ultra-deep Hi-C data found that A and B compartments alternate at the kilobase scale level (Harris et al., 2023). Another chromatin organizational unit is the topologically associating domains (TADs) that usually span between a few hundred Kbp to a few Mbp (Dixon et al., 2012). TADs set the stage for the finer organizational unit, the loops, which are chromatin interactions between regulatory elements or structural loops that connect CTCF-bound sites (Rao et al., 2014; Furlong and Levine, 2018). Enhancers and promoters within a TAD have a higher probability of interacting than if these regulatory elements were on different TADs. Nevertheless, there are examples of interactions spanning TAD boundaries, especially of developmentally important genes (Hung et al., 2024).

There are two prevailing models regarding the mechanism by which an enhancer interacts with the promoter of the gene it regulates: the classic looping model and the emerging phase separation model (Popay and Dixon, 2022). It is generally accepted that spatial proximity between an enhancer and a promoter is required, but the minimum distance of this interaction and how it relates to activity is highly debated. In support of the looping model and more specifically its instructive nature, targeted tethering of a looping factor allowed the *de novo* formation of a chromatin loop and activation of transcription (Deng et al., 2012). On the other hand, it has been observed mainly through super-resolution microscopy experiments that the timing of enhancer-promoter proximity is not always correlated with activation of gene expression (Alexander et al., 2019; Benabdallah et al., 2019). The latter findings, together with evidence that depletion of CTCF or cohesin, the main organizers of loop formation, does not lead to global transcriptional changes (Rao et al., 2017; Hsieh et al., 2022) have challenged the universality of the looping model. An alternative model, compatible with the above findings, is the phase separation model (Hnisz et al., 2017), according to which weak multivalent interactions between disordered regions in the activation domains of transcription factors and coactivators lead to condensates that separate from their surroundings, causing high local concentrations of activating factors, especially at super-enhancers. Containment of an enhancer and a promoter within the same condensate would allow gene activation without physical interaction through loop formation. Alternatively, the condensate could transiently interact with the regulatory elements and the gene locus to control gene bursting (Du et al., 2024). Clearly, more experiments are required to establish in a broader fashion the regulatory scenarios in which each of these two models for enhancer regulation applies.

## 4 Using machine learning to decipher the regulatory logic of enhancers

Machine learning-based approaches to discover patterns in genomics data are broadly categorized into non-neural network algorithms such as support vector machines (SVMs) and random forests (RFs) as well as the increasingly popular neural networks/deep learning approaches (Eraslan et al., 2019; Smith et al., 2023; Li et al., 2023). Neural networks utilize many layers of interconnected neurons to decipher hard-to-recognize patterns, hence the term “deep learning”. There are supervised neural network approaches comprised of the most popular convolutional neural networks (CNNs), the fully connected, the recurrent and the graph convolutional, as well as unsupervised methodologies such as autoencoders and generative adversarial networks, with the latter approaches mainly applied in single-cell genomics. In supervised learning, a model is obtained that takes features as input and delivers a prediction for the target variable, which is the desired output used to train the model. A machine learning algorithm is trained using a set of features, usually genomics data such as ChIP-seq or chromatin accessibility datasets that are split into three sets: the training set used for optimizing the parameters of the model, the validation set for evaluating the performance of the model and the test set for the assessment of the best model (Eraslan et al., 2019). The parameters of the network are randomly initiated and refined in an iterative fashion using batches of the training dataset for memory efficiency and prevention of overfitting, and the process is usually parallelized using graphical processing units (GPUs). The analyst can fine-tune different hyperparameters, such as the number of layers and the batch size, using the validation set before the final evaluation of the model using the test set. On the contrary, in unsupervised learning, unlabeled data are characterized by utilizing useful properties of the data. Unsupervised methodologies include autoencoders, which embed the data into a low-dimensional space and force the network to extract the most useful features. While autoencoders have found applications using bulk sequencing data such as extraction of gene signatures from RNA-seq data (Tan et al., 2017), they are also suited for single-cell applications such as improving clustering and denoising data (Wang et al., 2021). The generative adversarial networks offer a different approach consisting of two neural networks, a discriminator and a generator trained in parallel, with the generator creating realistic data and the discriminator classifying if a sample is real or created by the generator.

While simple linear “shallow learning” models like logistic regression can take care of standard tabular data, genomic sequences pose several challenges, such as local dependencies. For example, in classifying regions as bound or unbound by a transcription factor, representing enhancers as position-weighted matrices (PWMs) or k-mers with support vector machine approaches such as gkmSVM (Ghandi et al., 2016) may miss patterns where binding depends on cooperative interaction between multiple motifs with fixed spacing. In contrast, convolutional neural networks are perfectly suited to discern such complex dependencies (Eraslan et al., 2019; Smith et al., 2023; Li et al., 2023; Toneyan et al., 2022). They are composed of multiple layers, each scanning the sequence with several filters- PWMs and quantifying similarities between the filter and the sequence, followed by a non-linear activation function and pooling. Each subsequent layer composes the output of the previous layer that can be fed to a fully connected neural network that receives all information to perform the final prediction task. Applications of deep learning tools in genomics include prediction of regulatory elements such as enhancers, tf binding and assessing the effect of DNA variants. The main models for the above tasks and their salient features are presented in Supplementary Table S1. Early seminal applications of CNNs in genomics include DeepBind (Alipanahi et al., 2015), DeepSEA (Zhou and Troyanskaya, 2015) and Basset (Kelley et al., 2016), which were trained on large-scale chromatin accessibility or transcription factor binding data and were used to prioritize variants by predicting their effect on chromatin accessibility patterns. The DanQ algorithm (Quang and Xie, 2016) uses a hybrid architecture of CNNs and bidirectional long short-term memory recurrent networks that deal effectively with long-range dependencies. Another algorithm, the improved successor of Basset, Basenji (Kelley et al., 2018), uses dilated convolution to increase the receptive field, thus accommodating inputs of 131 Kbp again to effectively take into consideration long-range dependencies. Transformers, previously used in natural language processing applications, are particularly capable of handling pairwise interdependencies in DNA sequence data. The Enformer algorithm (Avsec et al., 2021) was the first to employ a combination of CNN and Transformer architecture and achieved higher accuracy compared to previously designed tools. A recent approach, BPNet, trained with high-resolution ChIP-exo data, is a state-of-the-art algorithm and its architecture contains a 10-layer CNN, with 64 filters per layer with dilated convolution, thus achieving a receptive field of 1,034 bp for any position in the genome (Avsec et al., 2021). In general, models that use a large input sequence (receptive field), such as Basenji and Enformer, can better predict long-range enhancers. Together with BPNet, these three models are the state-of-the-art in supervised learning for tasks related to deciphering the enhancer regulatory code (Toneyan et al., 2022). Finally, it is interesting to mention that the constant maturation of deep neural network algorithms has led to a recent milestone in enhancer biology, the construction of synthetic enhancers with the aid of deep neural network models such as DeepSTARR (de Almeida et al., 2022; de Almeida et al., 2024; Taskiran et al., 2024).

There are also deep learning tools dedicated to other applications in genomics, such as predicting the 3D architecture from DNA sequence as well as predicting DNA methylation states. Machine learning approaches such as Akita (Fudenberg et al., 2020), DeepC (Schwessinger et al., 2020), Orca (Zhou, 2022), Epiphany (Yang et al., 2023) and C. origami (Tan et al., 2023) have been used to predict the 3D genome organization from the linear sequence and/or 1D epigenomics experiments (Wang et al., 2024; Smaruj et al., 2025) (Supplementary Table S2). One major distinction between approaches is the type of input data, with some tools using only DNA sequence (Akita, DeepC, ORCA), while Epiphany inputs only epigenomics data. Sequence-based-only approaches cannot make accurate *de novo* predictions in different cell types, while epigenomics-only approaches usually require an array of different datasets to improve predictive power. Combining the above approaches, C. origami (Tan et al., 2023) is expected to achieve better predictive accuracy at the expense of possibly introducing uncertainties in model interpretation. Another discriminating characteristic of 3D predictive models includes the type of output, which is either a 2D contact map (Akita, Orca, C. origami) or predicted pixels directly from the 1D representation (DeepC, Epiphany), with the former possibly offering advantages as it makes use of the local correlation structure of contact map data (Smaruj et al., 2025).

Finally, tools such as DeepCpG (Angermueller et al., 2017) and MethylNet (Levy et al., 2020) based on CNNs have been used to predict DNA methylation states.

### 4.1 Model sharing and interpretation

Models are usually created using machine learning frameworks such as TensorFlow (Abadi et al., 2016), PyTorch (Adam et al., 2017) and Keras (Chollet, 2015), a user-friendly API that operates on frameworks like PyTorch. Models can be shared through repositories called model zoos available through the above frameworks as well as Kipoi (Avsec et al., 2019), a model zoo dedicated to genomics.

Although deep neural networks are often criticized as being “black boxes”, they can nevertheless be interpreted with various methodologies. For example, in perturbation-based methods, the input is modified and changes in the output are inspected. In the case of DNA sequence-based models, perturbations could involve a single nucleotide substitution (Alipanahi et al., 2015; Zhou and Troyanskaya, 2015). Nevertheless, the above approaches are computationally expensive, in contrast to attribution-based approaches such as saliency maps (Simonyan et al., 2013). The latter attribute a model’s intermediate network value to the input, with the magnitude of the score showing the amount of contribution. Two state-of-the-art approaches in model interpretation for transcription factor motif-fed algorithms include DeepLift (Shrikumar et al., 2016) and TF-MoDISco (Shrikumar et al., 2020). DeepLift decomposes the output of a neural network on a specific input by backpropagating the contributions of all neurons in the network to every feature of the input. In this way, it quantifies the importance of each nucleotide in a sequence for the model prediction. On the other hand, TF-MoDISco is suitable for motif interpretation and discovery and uses all neurons of a network to process sequence importance scores. In doing so, it clusters the most important nucleotides from different sequences into motifs.

## 5 Enhancer identification and machine learning tools in health and disease

Predicting enhancers and deciphering their regulatory code (Kim and Wysocka, 2023) could have far-reaching ramifications not only for discovering fundamental regulatory mechanisms (Kim et al., 2021) but also for diagnosing and treating various diseases (Karczewski and Snyder, 2018). A salient example of the former includes the application of chromBPnet, a convolutional neural network, in characterizing the regulatory syntax of enhancers that drive the reprogramming of human fibroblasts to pluripotent cells (Nair et al., 2023). ChromBPnet was used to infer putatively bound tf motifs that influence chromatin accessibility, thus offering insights on transcriptional regulators that drive cellular reprogramming. Moreover, genomics-based enhancer identification frequently in conjunction with machine learning tools has been used to subtype, stage and predict drug responses for various types of cancer such as colon, Ewing sarcoma and hematological malignancies (Akhtar-Zaidi et al., 2012; Riggi et al., 2014; Morgan and Shilatifard, 2015; Ntziachristos et al., 2016; Sur and Taipale, 2016; Morrow et al., 2018; Mack et al., 2019; Cejas et al., 2019; Shang et al., 2019; Yao et al., 2021; Zhu et al., 2024) and other conditions, such as heart failure (Spurrell et al., 2022) and Alzheimer’s disease (Berson et al., 2023).

Sepsis is another field of applicable but understudied enhancer discovery, as a condition characterized by important morbidity and mortality (Giamarellos-Bourboulis et al., 2024b), associated with a highly heterogenous but always dysregulated host immune response. Several sepsis subtypes have been identified among patients presenting with immunoparalysis (Cheng et al., 2016), those with macrophage activation-like syndrome (Karakike and Giamarellos-Bourboulis, 2019), as well as the novel category of patients with high interferon gamma and CXCL9 levels (Giamarellos-Bourboulis et al., 2024a). Other studies have applied unsupervised clustering to identify three or four distinct sepsis endotypes (Scicluna et al., 2017; Sweeney et al., 2018). Heterogeneity is addressed in other machine learning approaches, interrogating the transcriptome as conditioned by the pathogen or the predominating immune response (Komorowski et al., 2022); for example, bvnGPS2 has been trained with transcriptomic data to discriminate between bacterial and viral infections (Xie et al., 2024). Other deep learning models (Zhang et al., 2020; Davenport et al., 2016) trained with transcriptomic data from sepsis patients derived two classes of patients, with class 1 characterized by immunosuppression and higher mortality rates.

The large-scale application of epigenomics methodologies in sepsis that are needed to train machine learning algorithms is still at a rudimentary stage. MAGICAL (Chen et al., 2023), a hierarchical Bayesian framework, using single-cell RNA-seq and single-cell ATAC-seq data from peripheral blood mononuclear cells, was able to identify epigenetic circuit biomarkers distinctive of methicillin-susceptible or -resistant *Staphylococcus aureus* bloodstream infection. Nevertheless, there is an urgent need to produce large-scale epigenomics datasets from patients with sepsis, as epigenomics datasets, at least in other experimental systems (Dinh et al., 2020), have been found to have superior performance compared to transcriptomics data in stratifying patients and predicting responses to therapy.

Moreover, other immune response mechanisms, such as tolerance and resilience, are underrepresented in current subtyping efforts, and corresponding signatures are still missing (Shankar-Hari et al., 2024). Putative enhancers marked by H3K4me1 and H3K27ac that are activated in response to lipopolysaccharide-treated monocytes (Saeed et al., 2014) significantly overlapped sepsis-associated single nucleotide polymorphisms (SNPs) that were differentially expressed among two sepsis classes (Davenport et al., 2016). Moreover, there is evidence that histone modifications can act as a reservoir of epigenetic memory in immune tolerance and trained immunity (Netea et al., 2020). For a comprehensive review of epigenetic markers in sepsis, interested readers are referred to a recent review (Binnie et al., 2020). Finally, although promising immunotherapies have recently become available (Kyriazopoulou et al., 2021; Karakike et al., 2022), there is an urgent need for a deeper, holistic understanding of the immune dysregulation during sepsis and the development of novel biomarkers besides the classic markers already used in clinical practice (Karakike et al., 2024).

## 6 Discussion

Machine learning algorithms for genomics are evolving at a fast pace and as described above, have found applications in identifying enhancers, predicting their most crucial motifs and regulatory logic, as well as assessing the effect of variants on their function. In the latter application, state-of-the-art algorithms such as Enformer (Avsec et al., 2021) trained with large-scale structural (ChIP-seq, ATAC-seq) and functional epigenomics data such as massively parallel reporter assays (Arnold et al., 2013) and SELEX-like assays (Yan et al., 2021) could aid in assessing variants, for example, in immune conditions, such as sepsis where datasets are more scarce (Stankey and Lee, 2023). Nevertheless, machine learning algorithms are faced with challenges in their application, such as overfitting, meaning they do not generalize well to unseen examples. This could be due to the limited number of validated ground truth enhancers, the scarcity of large-scale training data beyond a small number of well-studied cell lines, and the presence of biological and technical variation in training datasets (Li et al., 2023).

The problem of data insufficiency could be ameliorated by using pre-trained self-supervised models adapted from the field of natural language processing, where models such as BERT (Devlin et al., 2019) and GPT (Brown et al., 2020) have achieved great success. For example, DNA-BERT (Ji et al., 2021) is a pre-trained bidirectional encoder that tokenizes the genome into k-mers, has cross-species transfer learning capabilities, and performs on par with other algorithms in tasks such as variant interpretation and transcription factor binding site prediction using only small amounts of task-specific labeled data. Other recently developed foundation models include Nucleotide Transformer (Dalla-Torre et al., 2025) and Alpha Genome (Avsec et al., 2025). Nevertheless, a comparison of pre-trained genomic language models with supervised tools such as Enformer did not find a clear advantage for self-supervised models in a variety of genomics tasks (Tang et al., 2025). Another challenge in clinical settings such as sepsis is that the presence of baseline datasets before overt disease onset (Garcia Lopez et al., 2024) is very rare. Perhaps in this scenario, machine learning algorithms could be used to impute missing datasets (Schreiber et al., 2023).

With more sophisticated and efficient algorithms trained with well-validated ground truth datasets, the stage is set for machine learning applications to delve into biological mechanisms and provide groundbreaking insights for the betterment of human health.
