# Supplementary material for: Machine learning tools for deciphering the regulatory logic of enhancers in health and disease
Source: Front Genet. 2025 Aug 13;16:1603687. doi: 10.3389/fgene.2025.1603687 (PMC12380740; doi:10.3389/fgene.2025.1603687)
Supplement: Supplementary file 1 [file Table1.docx]

| **Model** | **Input size** | **Model architecture** | **Tasks** | **Interpretation approach** | **Training datasets** | **Training cell types** | **Citation** |
| --- | --- | --- | --- | --- | --- | --- | --- |
| DeepBind | 101 bp | CNN | TF, variant | Example-based and perturbation-based | PBM, ChIP-seq, HT-SELEX | Multiple human, mouse | Alipanahi et al., 2015 |
| DeepSEA | 1 kb | CNN | DHS, histone, TF, variant | Model-based, Perturbation-based | DNaseI-seq, tf ChIP-seq | Multiple human | Zhou and Troyanskaya, 2015 |
| Basset | 600 bp | CNN | DHS, variant | Perturbation-based | DNaseI-seq | Multiple human | Kelley et al., 2016 |
| DanQ | 1 kb | CNN+BiLSTM | DHS, histone, TF, variant | Example-based | DNaseI-seq, tf ChIP-seq | Multiple human | Quang and Xie, 2016 |
| Basenji | 131 kb | CNN | DHS, histone, variant | Attribution-based | DNaseI-seq, histone ChIP-seq | Multiple human | Kelley et al., 2018 |
| Enformer | 196 kp | CNN+transformer | DHS, histone, TF, variant | Attribution-based | DNaseI-seq, ATAC-seq, ChIP-seq, CAGE | Multiple human, mouse | Avsec et al., 2021^a^ |
| BPNet | 1034 bp | 10-layer CNN | TF | Perturbation-based and attribution-based | ChIP-nexus | Mouse ESCs | Avsec et al., 2021^b^ |

BiLSTM: Bidirectional Long Short Term Memory

CNN: Convolutional Neural Network

PBM: Protein Binding Microarrays

Supplementary Table 1: Presented are some of the main deep learning models for predicting enhancers, tf binding and the effect of non-coding variants.
