# Supplementary material for: Machine learning tools for deciphering the regulatory logic of enhancers in health and disease
Source: Front Genet. 2025 Aug 13;16:1603687. doi: 10.3389/fgene.2025.1603687 (PMC12380740; doi:10.3389/fgene.2025.1603687)
Supplement: Supplementary file 2 [file Table2.docx]

| **Model** | **Input** | **Input size** | **Resolution** | **Model frameworks (Encoders-Long Range associations)** | **Data dimension conversion methodology for Decoder** | **Interpretation approach** | **Citation** |
| --- | --- | --- | --- | --- | --- | --- | --- |
| Akita | **DNA sequence-only** | 1 Mb | 2048 bp | CNN, dilated | 1D to 2D | ISM | Fudenberg et al., 2020 |
| DeepC |  | ∼1Mb | 5 kb | CNN, dilated, transfer learning | zig-zag stripe | Saliency | Schwessinger et al., 2020 |
| Orca |  | 1 Mb - 256 Mb | 4 kb - 512 kb | CNN, dilated, multilevel cascading | 1D to 2D | Multiplexed ISM | Zhou, 2022 |
| Epiphany | **Epigenomics-only**  (DNaseI-seq, Histone modifications, CTCF) | 1 Mb | 5 kb | CNN, bidirectional long short term memory, general adversarial network | zig-zag stripe | Saliency and SHAP | Yang et al., 2023 |
| C.origami | **DNA sequence and epigenomics** (ATAC-seq, CTCF) | ∼2Mb | 8192 bp | CNN, dilated, transformer | 1D to 2D | Attention weights and GRAM | Tan et al., 2023 |

ISM: In Silico mutagenesis; SHAP: SHapley Additive exPlanations; GRAM: Gradient-weighted Regional Activation Mapping

Supplementary Table 2: Presented are the main deep learning methodologies to predict 3D contact matrices from DNA sequences and/or 1D epigenomics data.
